# COVID-19 Related Sentiment Analysis Using State-of-the-Art Machine Learning and Deep Learning Techniques

**DOI:** 10.3389/fpubh.2021.812735

**Published:** 2022-01-14

**Authors:** Zunera Jalil, Ahmed Abbasi, Abdul Rehman Javed, Muhammad Badruddin Khan, Mozaherul Hoque Abul Hasanat, Khalid Mahmood Malik, Abdul Khader Jilani Saudagar

**Affiliations:** ^1^Department of Cyber Security, Air University, Islamabad, Pakistan; ^2^Information Systems Department, College of Computer and Information Sciences, Imam Mohammad Ibn Saud Islamic University (IMSIU), Riyadh, Saudi Arabia; ^3^Department of Computer Science and Engineering, Oakland University Rochester, Rochester, MI, United States

**Keywords:** healthcare, COVID-19, pandemic, sentiment analysis, Twitter, internet of things

## Abstract

The coronavirus disease 2019 (COVID-19) pandemic has influenced the everyday life of people around the globe. In general and during lockdown phases, people worldwide use social media network to state their viewpoints and general feelings concerning the pandemic that has hampered their daily lives. Twitter is one of the most commonly used social media platforms, and it showed a massive increase in tweets related to coronavirus, including positive, negative, and neutral tweets, in a minimal period. The researchers move toward the sentiment analysis and analyze the various emotions of the public toward COVID-19 due to the diverse nature of tweets. Meanwhile, people have expressed their feelings regarding the vaccinations' safety and effectiveness on social networking sites such as Twitter. As an advanced step, in this paper, our proposed approach analyzes COVID-19 by focusing on Twitter users who share their opinions on this social media networking site. The proposed approach analyzes collected tweets' sentiments for sentiment classification using various feature sets and classifiers. The early detection of COVID-19 sentiments from collected tweets allow for a better understanding and handling of the pandemic. Tweets are categorized into positive, negative, and neutral sentiment classes. We evaluate the performance of machine learning (ML) and deep learning (DL) classifiers using evaluation metrics (i.e., accuracy, precision, recall, and F1-score). Experiments prove that the proposed approach provides better accuracy of 96.66, 95.22, 94.33, and 93.88% for COVISenti, COVIDSenti_A, COVIDSenti_B, and COVIDSenti_C, respectively, compared to all other methods used in this study as well as compared to the existing approaches and traditional ML and DL algorithms.

## 1. Introduction

Coronavirus disease 2019 (COVID-19) has severely impacted the daily lives of individuals across the globe (1). People worldwide use online media to state their viewpoints and general feelings concerning this phenomenon that has assumed control over the world by storm (2, 3). Social media platforms like Twitter have experienced exponential growth in tweets related to the pandemic in a short period (4, 5). The social networking site Twitter is a commonly used online media platform. It provides real-time information related to ongoing events concisely and captures the emotions and thoughts of the people. During this pandemic, people use the online media platform Twitter to express their feelings, opinions, emotions, and thoughts related to the worldwide pandemic (6, 7). It rapidly spread throughout the world by an increasing number of corona cases in a short time (8). This disease has affected many countries, even the countries with hardly any or no infections because if someone is in close proximity to other people and one of them becomes affected, they will undoubtedly be impacted (9). According to Naseem et al. (1), the World Health Organization (WHO) declared COVID-19 a pandemic on January 30, 2020. All the biomedical experts worldwide are relentlessly trying to control the disease and find possible cures for this viral infection. To control this pandemic situation, vaccination is the most effective strategy to prevent the spread of COVID-19 disease all around the world (10, 11). Vaccination is the first and crucial step to stop the coronavirus outbreak. People with COVID-19 symptoms must isolate themselves and get examined, and all must get vaccinated.

During the isolation process, people express their feelings on social media; however, social media contains real-time and valuable information about COVID-19; still, data from social media might be useless or misleading at times. Sufferings get multiplied if they find misleading and depressing information on social media. With a new normal of “staying at home,” “work from home,” and “isolation time,” social networking media has been extensively used to share news, opinions, emotions and advice. Misinformation information is defined as trying to mystify/mislead others with false or irrelevant information, such as “eating bananas is a preventative against the COVID-19 coronavirus disease.” A person going through this disease passed through several physical and mental. This brings about the need to quickly apply logical strategies to comprehend informative data streams. Online Social media platforms such as Twitter and Facebook contain a lot of noisy data, so identifying informative content from large and noisy data is a challenging task, but after cleaning it, this noisy data captures human feelings and emotions, expression, and thoughts. When analyzed carefully, it conveys a lot about the present mood, attitude, and nature of a large human community.

The social media users are increasing with time because they depend on social media for informative content, and the volume of data is also increasing; this focused on the use of Natural Language Processing (NLP) with different algorithms of Artificial intelligence (AI) to extract meaningful information efficiently (12). NLP and its applications have had a significant impact on social media text analysis and classification; however, the challenges of determining a content's inherent importance using NLP-strategies, such as contextual phrases and words, ambiguity in text or speech, necessitate the use of ML-based algorithms (13–15).

In this study, we use Twitter data for sentiment analysis to identify public sentiments to investigate the increased fear associated with coronavirus. Many traditional approaches have been used to identify human behavior and nature, which presents the possibility of increasing analyses by quickly doing sentiment classification using NLP techniques. Sentiment analysis and classification of COVID-19 and other disaster-associated scenarios and keywords associated with the Twitter data analysis are essentially analyzed using the proposed methodology presented in this study outlines. This study focused on the tweets analysis and identified global people sentiments from February 2020 to March 2020. We use machine learning (ML) and deep learning (DL)-based classification methods primarily used in AI applications; however, the discussion and the comparison of tweets' sentiment classification mechanisms is one of the essential contributions of this research. Various studies in the past focused on the identification of sentiment from COVID-19 tweets but, less work is available for the identification of both the topic and sentiment considered together (16).

The tweets from February 2020 to March 2020 are collected using the Twitter API. The tweets' sentiments are categorized into three classes (positive, negative, and neutral) (1). This article focused on creating a new dataset rather than efficient categorization of users' sentiment. Therefore, we propose a new model to categorize the user's sentiments about COVID-19.

The main contributions of this study are as follows:

- Design a Transformation-based Multi-depth DistilBERT model for sentiment analysis of tweets to identify sentiments concerning coronavirus from tweets.
- Extract sentiment-related concise information from tweets to automatically learn features without human intervention.
- Present a broad comparison between existing ML and DL text classification methods and discuss the given baseline results. The proposed model outperformed on real-life datasets compared to all previously used methods.

The remainder of this article is organized as follows. Existing work related to COVID-19 sentiment analysis is presented in section 2. Section 3 provides a detailed explanation of the selected dataset. The proposed methodology is described in section 4. Experimental analysis and results are explained in section 5, and finally, the conclusion of this study is presented in section 6.

## 2. Literature Review

Since the outbreak of the coronavirus pandemic, researchers have discussed its origin, effects, and trends. This section presents the tweet sentiment analysis using different ML, DL, and NLP methods. Extracting meaningful information from noisy data is a challenging task. ML and DL techniques are necessary tools (17) to do this task. Twitter is one of the finest social media platforms for news collection (18). For sentiment analysis on India, 24,000 tweets regarding COVID-19 were crawled from Twitter (19). They just extracted the tweets related to COVID-19 and visualized the sentiments of people regarding this pandemic, and they did not perform experiments using ML techniques.

Another research focused on the topics and sentiments of people expressed on Twitter about COVID-19. They collected tweets about COVID-19 and labeled them as positive, negative, and neutral, then analyzed these tweets for sentiment classification using different feature sets and classifiers. This work used only one evaluation metric, which is accurate, and obtained the highest accuracy using the Bidirectional Encoder Representations from Transformers (BERT) model, which is 94.80%. Sometimes, we utilize only classification accuracy to assess our model's performance; nevertheless, this is insufficient to evaluate our model. We need to testify the model's performance using precision, recall, and F1-score along with accuracy (1).

Another research work focused on the psychological effect of COVID-19 to analyze the nature and prevailing mood of human behavior (20). It analyzed that people are in crisis due to coronavirus and increased anxiety levels because of COVID-19 news. Multiple studies show analysis about the industrial crisis and economic impact of the COVID-19 crisis across industries and countries (21). Over the past few years, sentiment analysis based on tweets has been utilized in numerous applications due to the large amount of data collected from various social media platforms (22). It includes Twitter, Facebook, Reddit, and YouTube. The analysis shows flaws in the collected information (23). Different ML and DL classifiers test the short and long text information. For evaluation of a short text, logistic regression, and Naive Bayes give average results of 74 and 91%, respectively, but in the case of long text testing, both the models performed very poorly (24). Recently, people have been heavily dependent on social media news, and they are conveying their viewpoints, emotion, and feelings about this novel virus *via* social media posting (25).

The recent COVID-19 studies rely on public opinion, emotion, and sentiment analysis in English social media posts from various social Critical Legal Studies (CLS). This study uses RNN models such as LSTM, BiLSTM, and SAB-LSTM to train and test the dataset. They have used the F1-score performance measure; based on this performance measure, SAB-LSTM achieved a higher F1-score when compared to LSTM, and BiLSTM Model for COVID-19 sentiment detection (26). Social media platforms such as Reddit allows healthcare service providers to collect data related to public opinions, which can be used for human behavior analysis and knowledge discovery. This study presents a generic approach based on NLP, which can extract the most critical topics about COVID-19 related comments (27). Zhang et al. (28) proposed a sentiment classification system to classify the text not at document level only at a sentence level. This approach extracts features related to public opinions and uses WordNet lexical method dataset to organize the opinion words. So this method categorized the elements under the relevant opinion sentences. The extracted features are scored according to the frequency in the reviews. The authors provide a summary based on features. They identified the most important features from the dataset and achieved the best F1-score of 83.6% using the BILSTM model. Mukherjee et al. (29) used the RNN model (bidirectional long short-term memory) to use NLP methods and utilize a bidirectional RNN to learn patterns of relations from textual data for sentiment analysis. There is much work done in tweet sentiment analysis using different DL and NLP approaches as mentioned in the study (30, 31). This study proposed a sentiment classification approach using DL and NLP models (12). NLP techniques are used for topic modeling to identify valuable topics related to coronavirus, while the LSTM model is used for classification using the sentiment-140 dataset (32). Another research gathered 1 million tweets to capture people's sentiments toward mask usage as a preventative strategy in the COVID-19 pandemic (33). They also utilized NLP to analyze the growth in the frequency of positive tweets. Our analysis finds the most common topics the public addresses in their posts concerning all preceding works. We also reported benchmarked findings for identifying tweet sentiments using NLP approaches. We compare the performance of COVID-19 tweet sentiment analysis using several ML and DL algorithms. We propose a valuable feature set with the goal of improving accuracy. The suggested system examines the sentiments of collected tweets for sentiment classification and extracts the most significant feature sets, which aids in improving classification outcomes when compared to the baseline technique. Table 1 presents the summary of the existing work.

**Table 1:** Summary of the existing work.

**Paper title**	**Methodology**	**Dataset**	**Results**
COVIDSenti: A Large-Scale Benchmark Twitter Data Set for COVID-19 Sentiment Analysis (34)	Proposed a COVIDSenti dataset and performed sentiment analysis using multiple classifiers	COVIDsenti	Achieved highest result using BERT with 94.80% accuracy.
Sentiment analysis of nationwide lockdown due to covid 19 outbreak: Evidence from india (19)	Visualize Sentiments on COVID-19 tweets	Crawled 24000 COVID-19 tweets	Extracts sentiments of Indian's about COVID-19
COVID-19 Public Sentiment Insights and ML for Tweets Classification (24)	Used Naive Bayes and Logistic Regression for sentiment classification	Created their dataset	Obtained 91% with Naive Bayes and 74% accuracy with logistic regression
Deep Sentiment Classification and Topic Discovery on Novel Coronavirus or COVID-19 Online Discussions: NLP Using LSTM Recurrent Neural Network Approach (12)	Used LSTM model for sentiment classification of COVID-19 tweets	Coronavirus Posts in Reddit Platform	LSTM model achieved an accuracy of 81.15%
Bidirectional Long Short-Term Memory Networks for Relation Classification (28)	Extract the most important features for sentiment classification using BILSTM	SemEval-2010 task 8 dataset	Achieved the best F1-score of 83.6% using BILSTM model

## 3. Dataset Selection

We used four real-world datasets for experimental analysis. The dataset is obtained through the Github repository (1) shown below to assess the classifier's classification performance. Table 2 provides an overview of the datasets utilized in this study.

**Table 2:** Overview of datasets.

**Dataset\Label**	**Positive**	**Negative**	**Neutral**	**Total**
COVIDSenti-A	1,968	5,083	22,949	30,000
COVIDSenti-B	2,033	5,471	22,496	30,000
COVIDSenti-C	2,279	5,781	21,940	30,000
COVIDSenti	6,280	16,335	67,835	90,000

**Figure 1:**
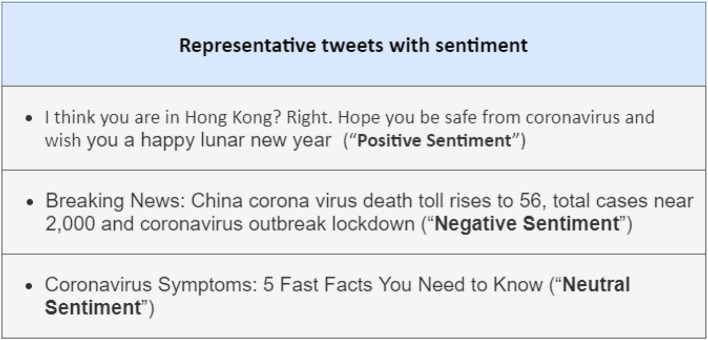
Representation of sentiment tweets in COVIDSenti.

The social media users indicate more negative sentiments than the positive sentiments corresponding to the COVID-19. The trend of negative sentiments concerning time shows that people using the Twitter platform are more intended toward negative sentiments than positive sentiments during the lockdown session from February 2020 to mid of March 2020. After mid of March 2020, the negative sentiment curve dropped because people followed SOP's and kept social distancing policies applied by the Government authorities. Naseem et al. (1) divided the COVIDSenti dataset into three parts for evaluation and generalization purposes: COVIDSenti_A, COVIDSenti_B, and COVIDSenti_C, which have three sentiments, positive, negative, and neutral for classification purposes. They labeled tweets as positive, negative, and neutral by following the Fellah and Bandi guidelines (35). The COVIDSenti dataset consists of two months of tweets fetch from Twitter using Tweepy, Python Twitter API library. Figure 1 represents the sentiment tweets in the COVIDSenti dataset.

**Selection Criteria:** COVIDSENTI is a two-month archive of tweets. Only English-language tweets were included in our search. The keywords guaranteed that the literary corpus focused on COVID-19 and related issues. Section 4.2 presents the keywords that are used to collect the tweets.

**COVIDSenti:** The COVIDSenti dataset consists of 90,000 unique tweets. These tweets are collected from 70,000 users from Twitter. A total of 2.1 million sentiment tweets from 2 months (February 2020 to March 2020) are collected. From these 90,000 unique instances, we have 6,280 positive sentiments, 16,335 negative sentiments, and 67,835 neutral sentiments for the classification task. The COVISenti dataset is further divided into three sub-datasets.

**COVISenti_A:** The COVIDSenti_A dataset consists of 30,000 tweets instances. From these 30,000 unique tweets, we have 1,968 positive sentiments, 5,083 negative sentiments, and 22,949 neutral sentiments for the classification task of the COVIDSent_A dataset. The COVIDSenti_A dataset contains tweets relevant to the action taken by government authorities to protect people from COVID-19. For example, “Coronavirus warning: UK threat increases as chief medical officers urge Government action.”

**COVIDSenti_B:** The COVIDSenti_B dataset consists of 2,033 positive sentiments, 5,471 negative sentiments and 22,496 neutral sentiments with overall 30,000 unique instances. COVIDSENTI-B dataset tweets mainly relate to four topics (COVID-19 disasters, keep social distancing, lockdown, and stay at home). COVISenti_B dataset shows tweets related to lockdown and stay at home even if one suffers from the disease. For example, “Stay Home If You Are Sick, or If you are sick stay home regardless of what you have.”

**COVIDSenti_C:** This dataset contains 2,279 positive sentiments, 5,781 negative sentiments, and 21,940 neutral sentiments, collectively 30,000 tweets from the Twitter platform related to three different topics (COVID-19 cases, stay at home, and outbreak). The COVIDSenti_C is a collection of tweets on COVID-19 cases, outbreaks, and stay-at-home advice. We find people's behavior from these topics and analyze that COVID-19 cases are increasing daily. For example, “Airport screenings for the Wuhan coronavirus increase around the world.”

## 4. Proposed Approach

**Figure 2:**
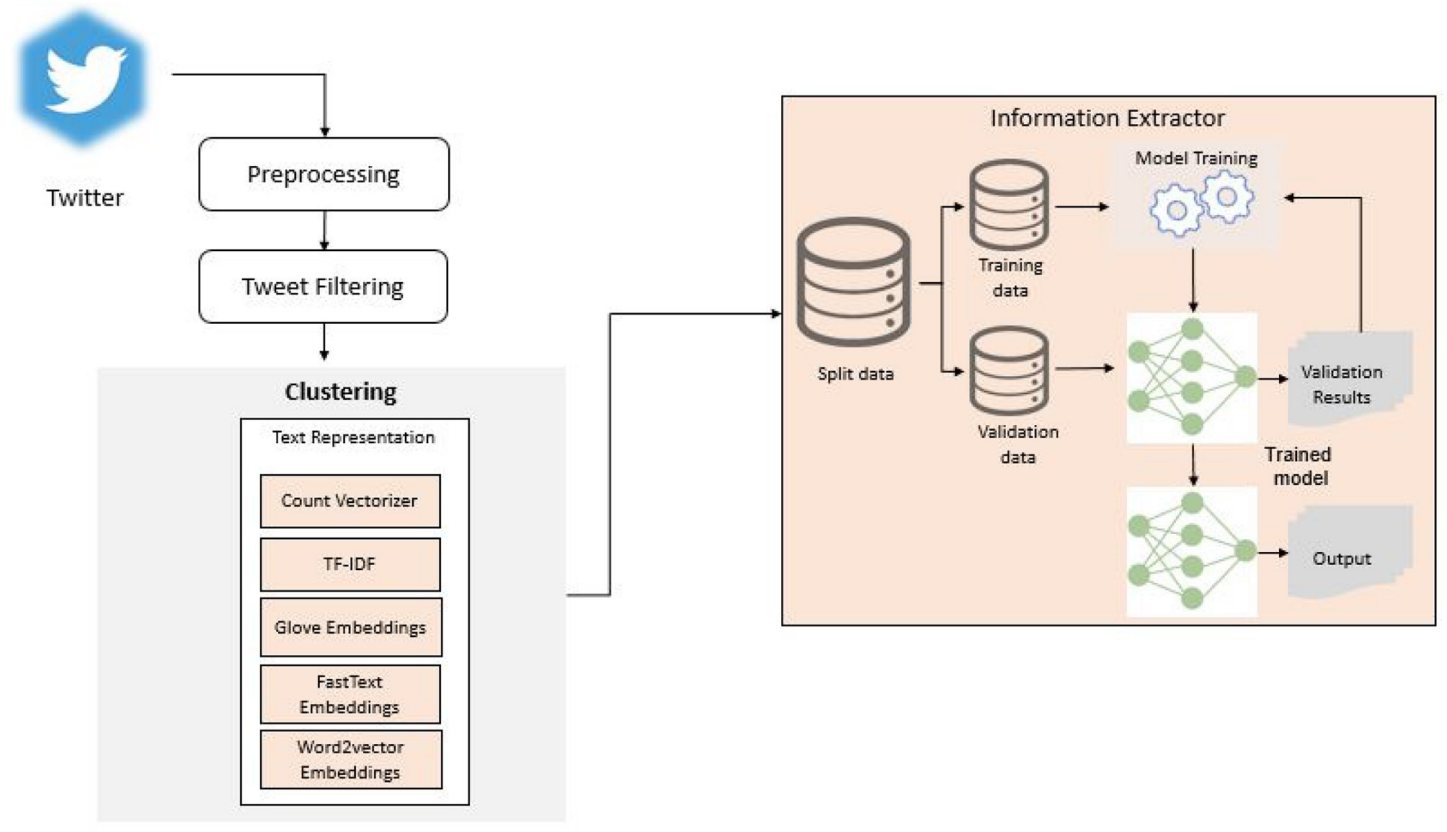
Overview of the proposed approach.

The proposed approach is divided into four phases: 1) pre-processing, 2) keyword trend analysis, 3) word embeddings for feature extraction, and 4) classification methods. The CovidSenti dataset is divided into two chunks, training and testing. We take care of the various factors of the dataset, such as over-fitting, noisy or small and large datasets. The main objective of this study is to evaluate the classification performance of state-of-the-art classifiers on the COVIDSenti dataset and then attempt to improve performance by extracting key features of tweets. The proposed technique classifies the CovidSenti dataset with higher accuracy and competently for the COVIDSenti dataset containing COVID-19 associated Twitter posts. Figure 2 demonstrates our proposed approach with each of the methods explained in the following figure.

### 4.1. Data Pre-processing

Information gathered from social networking media platforms is more often noisy and heterogeneous. We make the Twitter stream ready for exploratory analysis; the pre-processing step first changes the uppercase letters to lower case, then removes all the special characters, stop words, mentions, and URLs from the dataset tweets. For hashtag, first, it separates the total hashtags into tiny fragments as divided hashtags that positively affect the data clusters. A few hashtags are written using camel case, for example, “#StayHome,” which are easy to convert into the segment. Still, on the other hand, some hashtags that do not involve any camel case, e.g., “#stayhome,” a vast vocabulary is needed to discover the longest string suits in the hashtag. The pre-processing step uses a set of vocabulary of almost 70,000 English words to handle these challenges. Identifying informative content from a large and noisy dataset such as tweets is a challenging task. To achieve this, the following techniques are carried out inside the given order to enhance the text.

1. One common way to analyze COVIDSenti data is to calculate word frequencies to understand how often words are used in tweets. So, the first step is lemmatization that processes with the use of a vocabulary and morphological analysis of phrases and returns root words. We used lemmatization with the nltk method that converts a phrase to its base form, for example, “deaths” to “death” or “caring” to “care”).
2. The second step is to remove the stop words. It is the most suitable technique to overcome the noise from the textual tweets (such as “the,” “a,” “an,” “in”). Stop words can be filtered from the text to be processed, and it does no longer affect understanding of a tweet sentence's valence. We removed the most common stop-words that are present in a text, for example, “a,” “an,” “in,” and “the.”
3. Some time model understand the actual word as two different words because words are taken as case sensitive (i.e., COVID, covid). To avoid it, we convert all capital letters or words into the lower case. This method does not change the word's actual meaning or original word.
4. Contraction is a process of shortening the by through replacing or dropping letters with the aid of an apostrophe. Nowadays, people move to online media to connect with others *via* textual content or posts like Facebook, WhatsApp, Instagram, and Twitter. Many people communicate with each other; people mostly use shortened forms and abbreviations of words in their text. We used the contraction mapping method that drops the vowels from the words. Removal of contraction mapping is related to text standardization, and it is helpful while working with Twitter data in sentiment analysis.
  The main advantage of using textblob is its many capabilities like noun phrase extraction, pos-tagging, and sentiment analysis.
5. Part of speech is the essential step of pre-processing. It is essential to build parse trees, which might be utilized in constructing “most named entities are nouns” (NERS). It is also used to extract relations among words. Part of speech tagging is essential for building lemmatizers to return a phrase to its root form.
6. Twitter data is noisy, which affects the performance of a classifier, so the pre-processor eliminates URLs, @user_mentions. We remove alphanumeric or special characters and remove non-ASCII characters and numbers from our dataset because they do not help us detect sentiment. We also replace emojis with their corresponding reaction in text.
7. For hashtags, we eliminate the “#” symbol from the start of the phrase. We used tokenizer to split hashtags into appropriate words, for example, “#stayhomestaysafe,” tokenizer converted it into “stay,” “home,” “stay,” “safe.” Many words are concatenated in other words, and we also performed word-segmentation to achieve this.

### 4.2. Keyword Trend Analysis

We performed keyword trend analysis on our pre-processed corpus to find the most frequent words. The top 10 most commonly used keywords are “Coronavirus”, “Corona”, “COVID-19”, “Virus”, “Coronavirus Cases”, “Cases”, “Social distance”, “Coronavirus pandemic”, “Coronavirus Crisis”, and “Stay home” with 156,668, 87,661, 26,239, 22,289, 16,638, 10,269, 5,768, 3,125, 1,981 and 1,181 number of frequencies, respectively. We conclude that most people talk about coronavirus cases, the social distancing, COVID-19 outbreak, the coronavirus crisis, and stay-home.

To analyze the main topics across the dataset, we discover the topic distributions using Glove embeddings. We used Glove embeddings because it has an extensive vocabulary size. We can also find the words from COVIDSenti data that are not present in the Glove embeddings, such as contractions, misspelled words, concerted words, or emojis, which can decrease our model's performance. We discovered that most social media users discuss coronavirus cases, the coronavirus epidemic, social distance, the need to stay at home, the coronavirus crisis, and the crises caused by the coronavirus. Based on this analysis, we examine the distribution of the 15 topics across the corpora, in which topics 1 (“coronavirus”), 2 (“virus”), and 3 (“corona”) were the top three in the entire corpora.

### 4.3. Feature Extraction

In this study, count vectorizer, TF-IDF, and word embeddings techniques are used for feature extraction. The count vectorizer feature extraction technique is used to convert given tweets into a vector space, and it covers the tweets based on the most commonly used words (count) that frequently occur in the tweets. The count vectorizer creates a word matrix where every unique word represents the column of the matrix, and the selected text from the document represents the row of the matrix. In this way, we count the word in that particular text sample. We also used the term frequency-inverse document frequency (TF-IDF) feature extraction technique along with a count vectorizer. In this study, the TF-IDF is used for tweet analysis, and it has weighted features for execution boosting. The TF-IDF takes the TF and its corresponding IDF as a product to get the weights of features in a document. The length determines the TF of features in a single document. It is defined in Equation (1).


(1) $$\begin{array}{r} TF=\frac{count_{t,d}}{totalcount_{d}} \end{array}$$

In equation 1, the number of TF t in document d is represented by *count*_*t, d*_, while the overall number of terms in that document is represented by *totalcount*_*d*_. IDF thinks that the text's increase in term t will be more informative for model training. It can be defined as in equation 2.


(2) $$\begin{array}{r} idf^{{\;}^{\prime }}=i^{{\;}^{\prime }}/df_{t}^{{\;}^{\prime }} \end{array}$$

Where *i*′ is the total number of documents, and $df_{t}^{{\;}^{\prime }}$ is the number of documents that include the phrase t. When a term *t* frequently appears in many documents, IDF computes the weights of a phrase t low. For example, stop words have a lower IDF value. So finally, the TF-IDF can be defined as in equation 3.


(3) $$\begin{array}{r} tf-idf=tf_{t,d}*\log\left(idf\right). \end{array}$$

We also employed the word embeddings model for feature extraction, such as pre-trained models Word2Vec, Glove, and fastText embeddings with 300-D vectors. Furthermore, we used RCNN hybrid models for better sentiment classification tasks and reduced the over-fitting by decreasing bias because there are many variances. We also used transformer-based language models that have been widely used in the NLP research area. We used Multi-depth DistilBERT transformer-based model to pre-train a smaller general-purpose language representation model. We fine-tuned the Multi-depth DistilBERT model to achieve high performance on multiple tasks like better sentiment classification. Most of the previous work investigated that the used-ability of the distillation is only for building task-specific models. Still, the primary purpose is to decrease the size of the BERT transformer model. We decreased the BERT transformer model by 40% and retained its actual language understanding capabilities by 97%, and it was 60% faster than BERT.

These transformer-based language models (LMS) show how the training methods are used. We used unsupervised learning in the training process to generate an LM. The NLP models are trained on extensive data; these models attain more improved context word representations than conventional and non-contextual word representation methods because of the large data size.

### 4.4. Classification Methods

To analyze the classification performance on the COVIDSenti dataset, we used different ML and DL-based classifiers to measure the performance in the sentiment classification tasks.

We used XGBoost (eXtreme Gradient Boosting) in our analysis. XGB classifier is similar to the Gradient Boosting classifier. It consists of multiple trees, and it is a tree base model which is why it has gained lots of attention in the past few years. Many weak learners are working parallelly distinct to Gradient Boosting; XGboost gives a speed boost because of this technique. XGboost uses L1 and L2 regularization methods to control over-fitting, which are not present in Adaboost and Gradient Boosting techniques. XGBoost has a new feature of scalability so that it performs better in the distributed environment than a single system. At every iteration of XGBoost, we calculate some errors. We used this error to correct the previous prediction and optimize the loss function. Regularizer is utilized in the loss function to evaluate the classifier performance, which is defined by

(4) $$\begin{array}{r} X^{{\;}^{\prime }}\left(⊗\right)=L^{{\;}^{\prime }}\left(⊗\right)+\Omega \left(⊗\right). \end{array}$$

In equation (4), we train the parameters using ⊗, for the training Loss function, we used *L*′, and to measure the model's complexity, we used ω regularization. In this study, we set different parameters for the proposed XGBoost classifier. The XGB classifier is composed of 1,000 n_estimators with a 0.1 learning rate. Learning rate (LR) is used to handle the over-fitting. The XGB used a minimum of one child weight and a maximum depth of 6.

For DL-based models, we used the Conv1D-LSTM model. While using the DL classifier, we used Adam optimizer and L2 regularization. We trained the model on 50 epochs. Our Conv1D-LSTM consisted of four layers: the embedding layer with weights and vocab_size as input, then the Conv1D layer with a relu activation function. Next, we used the LSTM layer, and then at the end, the dense layer is used, also called an output layer. The softmax is employed as an activation function. In the experiments, we used a hybrid model named RCNN. We used this hybrid model for the sentiment classification task. RCNN model consisted of six layers in which one CNN layer with relu activation function, and then we used BiLSTM, an LSTM layer. After that, we used a dropout of 0.4 to control over-fitting; finally, we used a dense layer with softmax as an activation function. The RCNN uses Adam optimizer with verbose = None. We used the Multi-depth DistilBERT transformation model at the end of our experiments. The parameters of the Multi-depth DistilBERT model are presented in Table 3.

**Table 3:** The parameter settings of multi-depth DistilBERT.

**Value**	**Setting**
Model name	distilbert-base-uncased
Number of epochs	3
Batch size	80
Max sequence length	256
Learning rate	5e-05
Accumulation steps	4
Random seed	42

## 5. Experimental Analysis and Results

The main aim of this study is to evaluate the classification performance on COVIDSenti datasets and provide benchmarked results. This paper applies ML, DL, and hybrid methods to COVIDSenti datasets and examines their performance using several evaluation measures. The performance assessment metrics in this study include accuracy, precision, recall, and F1-score. These standard performance indicators have been carefully chosen to attest to the model's capacity to generate the best categorization performance. Following the experimentation procedure, the experimental outcomes are compared to state-of-art-methodologies.

### 5.1. Results

This work consists of different ML, DL, hybrid-based, and transformer-based models on four COVIDSenti datasets and analyzes their performance using the defined evaluation metrics. The proposed approach is used to gauge the performance in the sentiment classification task. The highlighted results in Tables 4–9 represent the highest achieved accuracy as compared to baseline results.

**Table 4:** Machine learning (ML) classifiers accuracy using count vectorizers.

**Model dataset**		**COVIDSenti**	**COVIDSenti_A**	**COVIDSenti_B**	**COVIDSenti_C**
	**Proposed model/Accuracy**				
Count vectorizer	KNN	79.21%	79.65%	77.83%	77.75%
	LR	90.03%	87.75%	86.76%	86.04%
	Ensemble	88.75%	88.51%	87.08%	86.36%
	**XGB**	**89.81%**	**88.71%**	**88.03%**	**87.07%**

*The bold ones represent better results*.

**Table 5:** Comparison of proposed deep learning (DL) Classifiers accuracy with baseline using word embeddings.

**Model dataset**		**COVIDSenti**	**COVIDSenti_A**	**COVIDSenti_B**	**COVIDSenti_C**
**Existing models/Accuracy**					
Word2vec	BiLSTM	76.09%%	76.05%	74.05%	74.09%
Glove	BiLstm	77.01%	76.08%	72.09%	74.03%
DCNN- (Glove+ CNN)		86.09%	83.04%	83.02%	86.04%
**Proposed model/Accuracy**					
**Conv1D-LSTM + Glove**		**87.06%**	**86.10%**	**84.44%**	**86.90%**
Gain		**0.97%**	**3.06%**	**1.42%**	**0.86%**

*The bold ones represent better results*.

**Table 6:** Comparison of proposed hybrid model accuracy with baseline.

**Model dataset**	**COVIDSENTI**	**Covidsenti_A**	**Covidsenti_B**	**CovidSenti_C**
**Existing models/Accuracy**				
IWV	77.01%	76.03%	74.09%	73.05%
HyRank	88.01%	85.04%	86.05%	87.07%
**Proposed model/Accuracy**				
**RCNN**	**95.40%**	**92.90%**	**92.93%**	**93.27%**
Gain	**7.39%**	**7.86%**	**6.88%**	**6.20%**

*The bold ones represent better results*.

**Table 7:** Comparison of proposed ML classifiers accuracy with baseline using term frequency-inverse document frequency (TF-IDF).

**Model dataset**		**COVIDSenti**	**COVIDSenti_A**	**COVIDSenti_B**	**COVIDSenti_C**
**Existing models/Accuracy**					
TF-IDF	SVM	84.05%	83.09%	83.00%	82.08%
	RF	84.01%	83.06%	82.08%	82.01%
	NB	77.03%	76.05%	75.00%	73.01%
	DT	79.04%	78.01%	76.08%	75.03%
**Proposed model/Accuracy**					
	**XGB**	**88.46%**	**88.31%**	**87.41%**	**86.03%**
	Gain	**4.41%**	**5.22%**	**4.41%**	**3.95%**

*The bold ones represent better results*.

**Table 8:** Comparison of proposed ML classifiers accuracy with baseline using word embeddings.

**Model dataset**			**COVIDSENTI**	**Covidsenti_A**	**Covidsenti_B**	**CovidSenti_C**
**Existing models/Accuracy**						
Word2Vec	RF		76.09%	76.04%	73.02%	75.03%
	DT		76.06%	76.02%	74.05%	74.01%
Glove	RF		72.06%	71.06%	70.02%	72.02%
	DT		70.01%	69.03%	68.09%	69.04%
FastText	SVM		81.05%	80.01%	79.02%	78.03%
	NB		73.05%	73.02%	74.05%	72.01%
	RF		84.05%	82.03%	84.01%	80.02%
**Proposed model/Accuracy**						
Fine tuned FastText		XGB	80.00%	78.33%	77.61%	76.30%
Fine tuned Word2Vec		XGB	79.71%	78.33%	77.85%	76.05%
**Fine tuned Glove**		**XGB**	**79.19%**	**78.39%**	**78.23%**	**76.05%**
Loss			**4.05%**	**3.64%**	**5.78%**	**3.97%**

*The bold ones represent better results*.

**Table 9:** Comparison of proposed transformer based model accuracy with baseline models.

**Model dataset**	**COVIDSenti**	**COVIDSenti_A**	**COVIDSenti_B**	**COVIDSenti_C**
**Existing models/Accuracy**				
distilBERT	93.09%	93.07%	92.09%	92.06%
BERT	94.08%	94.01%	93.07%	92.02%
XLNET	93.03%	92.04%	91.04%	92.00%
ALBERT	92.09%	91.04%	92.00%	91.01%
**Proposed model/Accuracy**				
**Multi-depth DistilBERT**	**96.66%**	**95.22%**	**94.33%**	**93.88%**
Gain	**2.58%**	**1.21%**	**1.26%**	**1.82%**

*The bold ones represent better results*.

#### 5.1.1. COVIDSenti

**Figure 3:**
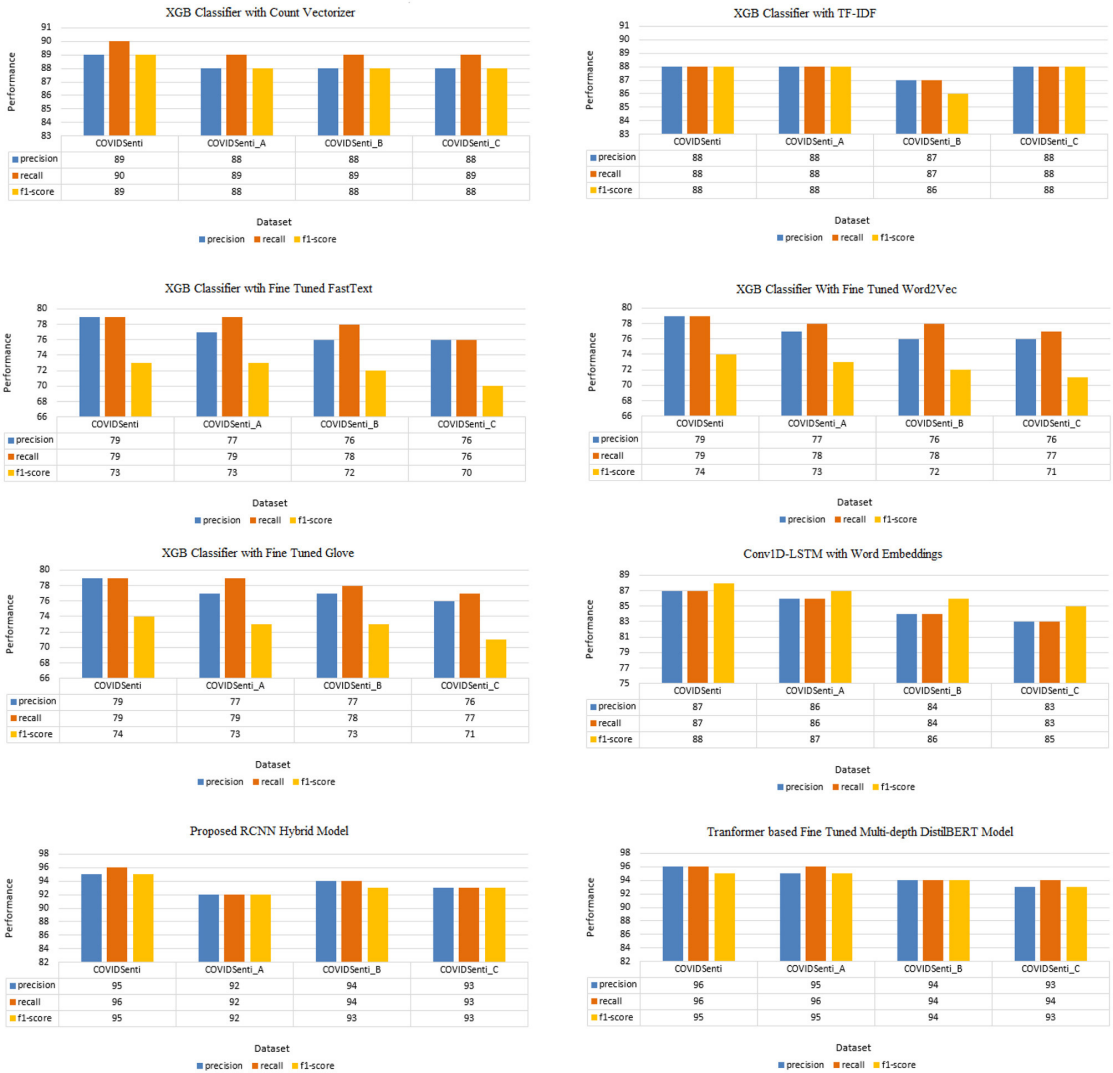
Precision, Recall, and F1-score of classifiers used in proposed approach (percentage results).

Figure 3 and Tables 4–9 present the results achieved on the COVIDSenti dataset for tweet sentiment classification. In the case of the count vectorizer feature extraction technique, for sentiment classification on the COVIDSenti dataset, the XGB model attains the best accuracy of 89.81%, other ML techniques, such as KNN, LR, and Ensemble model, achieve the accuracy of 79.21, 89.03, and 88.75%, respectively. The xgb model achieves the highest accuracy of 89.81% with 89% precision, 90% recall, and 89% F1-score compared to KNN, LR, and Ensemble model.

While using the TF-IDF feature extraction technique for sentiment classification again, the xgb model outperforms other ML models, such as SVM, RF, NB, and DT with the highest accuracy of 88.46% with 88% precision, 88% recall, and 88% F1-score. We also used different word embedding techniques, such as FastText, Word2Vec, and Glove with different ML classifiers. We fine-tuned FastText with a Glove, resulting in 80% accuracy on the COVIDSenti dataset. We also measure other evaluation metrics like precision, recall, F1-score, 79% precision for the COVIDSenti dataset, 79% recall, and 73% F1-score. While working with the Word2Vec word embedding technique, we used the xgb model and achieved an accuracy of 79.71% which is the highest accuracy compared to other Word2Vec models (RF with 79.09% and DT with 76.06%). Along with the accuracy, we measure the precision, recall, and F1-score of the xgb model, which is 79, 79, and 74%, respectively. Finally, we employ the Glove word embedding technique with the ML model. We fine-tuned Glove with the xgb model and gained an accuracy of 79.19% with the precision of 79%, recall of 79%, and F1-score of 74%. After ML with word embeddings, we employ DL with word embeddings. The proposed model Conv1D-LSTM with Glove gains an accuracy of 87.60%. The best precision, recall, and F1-score were gained from the Conv1D-LSTM classifier, 87, 87, and 88%, respectively.

We used the RCNN hybrid model for the sentiment classification task, and it performs very well on the COVIDSenti dataset. The best accuracy of hybrid models is achieved from the RCNN model, which is 95.40%. RCNN model shows a better 95, 96, and 95% in terms of precision, recall, and F1-score. Finally, we used a transformer-based language classifier which shows the overall highest accuracy of 96.66% with the highest precision of 96% also with 96% recall and 95% accuracy.

#### 5.1.2. COVIDSenti_A

The results achieved on the COVIDSenti_A dataset are presented in Tables 4–9 and plotted in Figure 3.

We used the count vectorizer feature extraction technique to classify sentiments first. The highlighted XGB model gains the highest accuracy of 88.71% compared to other ML models: KNN, LR, and Ensemble, 79.65, 87.75, and 88.51%, respectively. We also measure the other evaluation metrics (precision, recall, and F1-score) for the XGB classifier. The precision score of the XGB model for the COVIDSenti_A dataset is 88%, recall score is 89%, and the F1-score score is 88%. We also used different ML classifiers with the TF-IDF feature extraction technique. The XGB classifier on the COVIDSenti_A dataset exhibits the highest accuracy of 88.31% with 88% precision, 88% recall, and also 88% F1-score as compared to other ML models such as SVM, RF, NB, and DT. We also apply different word embedding techniques (FastText, Word2Vec, and Glove) on the COVIDSenti_A dataset.

In the case of FastText word embedding with XGB classifier on COVIDSenti_A, we achieve 78.33% accuracy with 77% precision, 79% recall, and 73% F1-score. We also used Word2Vec embedding with XGB classifier on COVIDSenti_A dataset. XGB classifier with Word2vec word embedding achieves an accuracy of 78.33% which is the highest accuracy compared to other word2vec techniques with ML classifiers. The XGB classifier with Word2Vec gains a precision score of 77%, recall score of 78%, and F1-score of 73%. Finally, we used Fine-tuned Glove word embedding with XGB classifier, which achieves the highest accuracy compared to Word2vec and Glove word embeddings with ML. The fine-tuned Glove with XGB classifier achieves an accuracy of 78.39% on the COVIDSenti_A dataset. We also measure other performance metrics to testify to the model's capability. Other performance metrics are precision with 77%, recall with 79%, and F1-score with 73%.

After ML word embedding, we move on to DL word embedding for better sentiment classification. While working with DL classifiers with word embeddings, we apply Conv1D-LSTM with the Glove word embedding technique. Conv1D-LSTM with Glove word embedding achieves the best accuracy of 86.10% with other performance metrics such as precision, recall, and F1-score. The Conv1D-LSTM model observes the maximum F1-Score, precision, and recall, i.e., 86%, 86%, 87%, respectively, while having a maximum accuracy of 86.10%. We propose a hybrid model named RCNN. The best performance is achieved on the COVIDSenti_A dataset in terms of accuracy, precision, recall, and F1-score by the RCNN model with a maximum accuracy of 92.90% and maximum precision-recall, and F1-score of 92, 92, and 92%, respectively, as compared to other hybrid models. Finally, we proposed a Multi-depth DistilBERT transformer-based model, which performed very well on the COVIDSenti_A dataset and all other methods on the COVIDSenti_A dataset. The maximum accuracy on COVIDSenti_A using Multi-depth DistilBERT is 95.22% with precision, recall, and F1-score of 95%, 96%, 95%, respectively.

#### 5.1.3. COVIDSenti_B

Figure 3 and Tables 4–9 depict the sentiment classificaition results in the COVIDSenti_B dataset by different ML, DL, Hybrid models, and transformer based models. First, we used the count vectorizer feature extraction technique to extract useful features and pass them to models for sentiment classification tasks. We used the XGB model for sentiment classification on the COVIDSenti_B dataset. The XGB model obtains the maximum accuracy of 88.03% compared to other ML models such as KNN, LR, and Ensemble, which acquire 79.65, 87.75, and 88.51% accuracy. The best precision, recall, and F1-score on COVIDSenti_B are gained from the XGB classifier, 88, 89, and 88%, respectively.

Another feature extraction technique is used with ML classifiers for sentiment classification. Features are extracted using TF-IDF, and then we apply ML classifiers on these features for COVIDSenti_B dataset sentiment classification. We pass these TF-IDF features to the XGB model for better classification results, and XGB shows the accuracy of 87.41% on the COVIDSenti_B dataset with better precision, recall, and F1-score of 87, 87, and 86%, respectively. We used three different word embedding techniques with ML models to reduce the computational cost. First, we used Fine-tuned Glove with XGB ML model, which obtains an accuracy of 78.23% which is quite well as compared to other Word2Vec and Glove word embedding in Table 8. With the best accuracy of 78.23%, the Fine-tuned Glove with the XGB ML model gets the precision, recall, and F1-score of 77, 78, and 73%, respectively.

Second, we used the Word2Vec word embedding technique using the XGB ML model. The fine-tuned Word2Vec with XGB model gets the accuracy of 77.85% on the COVIDSenti_B dataset with a precision score of 76%, recall 78%, and F1-score of 72%. In the end, we used fine-tuned FastText word embedding with an XGB ML classifier. The fine-tuned FastText word embedding with XGB ML classifier gains less accuracy of 77.61% compared to proposed Glove and Word2Vec embeddings methods. The precision, recall, and F1-score of fine-tuned FastText word embedding with XGB ML classifier is 76, 78, and 72%, respectively.

We used Conv1D-LSTM with Glove word embedding for better results, so we used DL with word embeddings to achieve the highest results for sentiment classification. For this purpose, we train the Conv1D-LSTM model on the COVIDSenti_B dataset and get the evaluation result of 84.44% with other different performance metrics. The other performance metrics are precision, recall, and F1-score with 84, 84, and 86%, respectively. We also proposed a hybrid model by adding multiple layers of LSTM, BiLSTM, and CNN for better classification on the COVIDSenti_B dataset. To achieve better classification on the COVIDSenti_B dataset, RCNN hybrid model gets 92.93% with better precision, recall, and F1-score. The precision, recall, and F1-score of the RCNN model are 94, 94, and 93%, respectively. Finally, we apply a transformer-based model named Multi-depth DistilBERT on the COVIDSenti_B dataset, which gives an accuracy of 94.33% with 94% precision, 94% recall, and 94% F1-score. F1-score of 94% shows that this model performed very well on the COVIDSenti_B dataset.

#### 5.1.4. COVIDSenti_C

The results of the COVIDSenti_C dataset are shown in Tables 4–9 and plotted in Figure 3. We used different ML, DL, hybrid models, and transformer-based models for COVIDSenti_C dataset classification. We used the count vectorizer technique to extract essential features from the COVIDSenti_C dataset as shown in Table 4. When we extract essential features, we send these features to ML models to perform sentiment classification tasks. We used KNN, LR, Ensemble, and XGB models for sentiment classification on the COVIDSenti_C dataset. For sentiment classification in the COVIDSenti_C dataset, the XGB model obtains the best accuracy of 87.07%. Other conventional techniques, KNN classifier, LR classifier, and Ensemble classifier, get the accuracy of 77.75, 86.04, and 86.36%, respectively. XGB classifier shows a proficient gain of 88, 89, and 88% in terms of precision, recall, and F1-score, respectively, compared to other models.

Furthermore, we apply another technique to extract important features for better results. We extract important features using TF-IDF and send those features to multiple ML classifiers for classification purposes. Finally, the XGB classifier using these TF-IDF features delivers the best accuracy of 88.31% on the COVIDSenti_C dataset. The precision, recall, and F1-score of the XGB model with 88.31% accuracy are 88, 88, and 88%, as shown in Figure 3. The computational time plays an important role during the model's training process, so to reduce computational time, we apply different word embeddings to ML and DL models. For ML, first, we apply FastText with the XGB model. We fine-tuned FastText word embedding and passed this word embedding to the XGB model to reduce computational time, which results in 76.30% accuracy, 76% precision, 76% recall, and 70% F1-score. Less F1-score indicates that we need to improve the classification result. The second embedding technique to reduce computational time is Word2Vec. While using fine-tuned Word2Vec embeddings with the XGB model, we achieved better accuracy, 76.05%, and quite well precision, recall, and F1-score, which is 76, 77, and 71%. The last and essential word embedding technique we used in this study is Glove. While using fine-tuned Glove with the XGB classifier again, we obtained an accuracy of 76.05% with the same precision, recall, and F1-score of 76, 77, and 71%. We moved to DL with word embeddings; first, we used Conv1D with LSTM and Glove. This proposed model worked very well on the COVIDSenti_C dataset and gave the accuracy of 86.91% which is the highest accuracy of the COVIDSenti_C dataset as shown in Table 5 and also gives the best precision, recall, and F1-score of 83, 83, and 85% shown in Figure 3.

Furthermore, we propose our hybrid model, which performed very well on the COVIDSenti_C dataset. The proposed RCNN model achieves the best accuracy of 93.27% which is the highest accuracy in the COVIDSenti_C dataset is shown in Table 6. The evaluation metrics include precision, recall, and F1-score with 93% precision, 93% recall, and 93% F1-score. To cover all the methods with better classification results, we proposed a transformer-based language model that achieves the highest accuracy compared to all other methods used with COVIDSenti_C. The Multi-depth DistilBERT gives the highest accuracy of 93.88%, which is the highest accuracy of the COVIDSenti_C dataset compared to all other methods applied on the COVIDSenti_C dataset. It also gives the highest precision, recall, and F1-score of 93%, 94%, and 93% on the COVIDSent_C dataset.

### 5.2. Comparative Analysis With Baseline Approach

To assess the performance of the suggested strategy, we compare our experimental findings to those of the state-of-the-art approach (1), whose experimental circumstances are identical to those used in our work. The baseline approach used only accuracy as an evaluation metric to testify classifier's ability. As compared to baseline proposed approach used precision, recall, and F1-score along with accuracy metric. The proposed framework analyzes tweets' sentiments for sentiment classification and extracts the most important features that help increase the classification results compared to the baseline approach. Furthermore, we proposed a fine-tuned transformer-based Multi-depth DistilBERT model that achieved the highest results in terms of accuracy as compared to all the baseline results. The comparative results are reported in Tables 4–9. To achieve the higher performance for ML classifiers, we used different conventional methods: count-vectorizer, TF-IDF, different word embeddings based models like Word2Vec, fastText, and Glove. The results of count-vectorizer-based classification are shown in Table 4.

When using count vectorizer with KNN, logistic regression, ensemble method, and XGB ML classifiers on COVIDSenti datasets, the count vectorizer with XGB classifier exhibited better performance on the COVIDSENTI dataset than other ML classifiers(KNN, LR, and Ensemble). The classification accuracy of the XGB model using the count vectorizer method is 89.81% on COVIDSenti, 88.71% on COVIDSenit_A, 88.03% on COVIDSenit_B, and 87.07% on COVIDSenti_C, respectively.

In addition, we conducted a comparison using TF-IDF-based classification. The obtained results are shown in Table 7. On all COVIDSenti datasets, the TF-IDF was used in conjunction with the SVM, RF, NB, DT, and XGB models. Compared to other baseline ML classifiers, the TF-IDF approach with XGB classifier performs better on the COVIDSENTI-A dataset, COVIDSENTI-B dataset, COVIDSENTI-C dataset, and COVIDSENTI dataset. The classification accuracy of the XGB model using the TF-IDF method is 88.46% with 4.41% gain on COVIDSenti, 88.31% accuracy with 5.22% gain on COVIDSenit_A, 87.41% accuracy with 4.41% gain on COVIDSenit_B, and 86.03% accuracy with 3.95% gain on COVIDSenti_C, respectively. For the comparison of ML with word embeddings, we employed different word embeddings techniques like FastText, Glove, and Word2Vec with various ML classifiers: RF, DT, SVM, NB, and XGB as shown in Table 8. In the case of word2vec embedding, we fine-tuned word2vec with an XGB classifier, which shows better performance (79.17, 78.33, 77.85, and 76.07% on COVIDSENTI, COVIDSENTI-A, COVIDSENTI-B, and COVIDSENTI-C, respectively) as compared to baseline ML models with word2vec embeddings.

Furthermore, we fine-tuned the Glove with an xgb classifier for Glove word embedding, which outperforms the baseline Glove embedding with different ML classifiers. The xgb model achieves the highest accuracy of 79.19% on COVIDSenti, 78.39% on COVIDSenti_A, 78.23% on COVIDSenti_B, and 76.05% on COVIDSentiC as compared to baseline ML models. We detect a decrease in accuracy for the proposed FastText embedding model that is minor when compared to the yield inaccuracy for other word embedding techniques. We get the loss in terms of accuracy while comparing the proposed DL classifier with the word embedding approach to the baseline FastText approach, which is 4.05% for COVIDSenti, 3.64% for COVIDSenti_A, 5.78% for COVIDSenti_B, and 3.97% for COVIDSenti_C datasets. To compare DL-based classifiers, we apply various word embeddings models like Glove and 1D convolutional neural network (1DCNN) with LSTM, where Glove is utilized for word representations. The comparative results of DL-based classifiers are presented in Table 5 with baseline, Word2Vec, and Glove with DL classifiers on all three COVIDSenti datasets. Compared to baseline word embedding-based classifiers, our proposed approach Conv1D-LSTM with Glove showed better performance (87.60% with 0.97% gain, 86.10% with 3.06% gain, 86.44% with 1.42%, and 86.90% with 0.86% accuracy gain on COVIDSenti, COVIDSenti_A, COVIDSenti_B, and COVIDSenti_C).

In Table 6, we also compare hybrid models to baseline models such as the hybrid ranking model and IWV model. The baseline approach used a hybrid ranking model that includes sentiments and context of Twitter posts for Twitter sentiment analysis (36). The comparative results with baseline Hybrid ranking and IWV model are shown in Table 6. Notice that the proposed hybrid model (RCNN) outperforms the baseline Hybrid ranking model and IWV model with the performance score of 95.40% with 7.39% accuracy gain, 92.90% with 7.86% accuracy gain, 92.93% with 6.88% accuracy gain, and 93.27% with 6.20% accuracy gain on COVIDSenit, COVIDSenti_A, COVIDSenti_B, and COVIDSenti_C, respectively.

Table 9 shows the final comparison of transformer-based models. The previous study fine tuned the transformer-based models such as BERT, DistilBERT, XLNET, and ALBERT. Compared to previously fine-tuned transformer-based models, we fine-tuned the Multi-depth DistilBERT model that performed very well compared to all other transformer-based models and all the other approaches used before in this study. Experiments prove that the proposed Multi-depth DistilBERT model achieves better accuracy of 96.66% with 2.58% gain on COVIDSenti, 95.22% with 1.21% gain on COVIDSenti_A, 94.33% with 1.26% gain on COVIDSenti_B, and 93.88% with 1.82% gain on COVIDSenti_C, respectively.

## 6. Conclusion

Since the outbreak of the COVID-19 pandemic and with a new normal of staying at home, working from home, and “isolation time,” social networking media has been extensively used to share news, opinions, emotions, advice; however, most of the data on social media are irrelevant and do not belong to the actual scenario. This study proposed an approach to deal with the Twitter sentiment using the COVIDSenti dataset. We evaluate ML and DL classifiers using novel feature extracting methods that automatically learn features without human interference. We observed that people follow government policies and Standard Operating Procedures (SOPs) and began to favor lockdown and keep social distancing in March 2020, but the order by the government is in February 2020. There is much misinformation on social media; therefore, health organizations need to develop a stable system for detecting coronavirus precisely to preclude the spread of fake news. The proposed approach performed very well on the given dataset and showed higher accuracy when compared to similar state-of-the-art studies. In future work, we plan to analyze public sentiments toward other essential topics, such as government response to the pandemic situation, healthcare facilities by government, offline examination, and mental health by using DL algorithms to increase their performance on the dataset. One limitation of this work is that it is specific and does not look at the mood and emotions of the people. Further work can be done on the detection of mood-based sentiment analysis.

## Data Availability Statement

The datasets presented in this study can be found in online repositories. The names of the repository/repositories and accession number(s) can be found in the article/supplementary material.

## Author Contributions

ZJ, AJ, and AA: conceptualization. AA: data curation. AJ: formal analysis, investigation, methodology, and software. AS: funding acquisition. KM, AS, MB, and MA: project administration. ZJ, MB, and MA: resources. KM, AS, AJ, KM, and AS: supervision. AA and ZJ: validation. AA, KM, and AS: visualization. MB, MA, and ZJ: writing–review and editing. All authors contributed to the article and approved the submitted version.

## Conflict of Interest

The authors declare that the research was conducted in the absence of any commercial or financial relationships that could be construed as a potential conflict of interest. The handling editor declared a past co-authorship with several of the authors AJ and ZJ.

## Publisher's Note

All claims expressed in this article are solely those of the authors and do not necessarily represent those of their affiliated organizations, or those of the publisher, the editors and the reviewers. Any product that may be evaluated in this article, or claim that may be made by its manufacturer, is not guaranteed or endorsed by the publisher.
